# Body Composition Changes During GLP-1 Receptor Agonist Therapy in Pediatric Obesity: A Pilot Study

**DOI:** 10.3390/metabo16070460

**Published:** 2026-07-01

**Authors:** Bogdan Mihai Pascu, Irina Bojoga, Anca Bălănescu, Paul Cristian Bălănescu, Ioan Gherghina

**Affiliations:** 1 Pediatric Department, Faculty of Medicine, University of Medicine and Pharmacy “Carol Davila”, 030167 Bucharest, Romania; 2 National Institute for Mother and Child Health “Alessandrescu-Rusescu”, 020395 Bucharest, Romania; 3 Yuno Clinic, EASO COM Obesity Center, 020459 Bucharest, Romania; 4 Endocrinology and Diabetes Department, “Elias” University Emergency Hospital, 011461 Bucharest, Romania

**Keywords:** GLP-1 receptor agonist, pediatric obesity, body composition, muscle preservation, bioimpedance

## Abstract

**Highlights:**

**What are the main findings?**

- Weekly injectable semaglutide therapy in children with obesity significantly reduced BMI Z-score and fat mass over a median follow-up of 5 months, while fat-free mass index remained stable.
- Despite non-significant changes in absolute muscle mass, predicted muscle mass percentage increased significantly, suggesting selective fat loss with lean mass preservation.

**What are the implications of the main findings?**

- GLP-1 RA therapy may exert a muscle-sparing effect in adolescents with obesity, supporting its safety for use during important developmental periods.
- These pilot findings justify larger prospective studies with body composition as a primary endpoint to confirm the role of GLP-1 RAs in pediatric obesity management.

**Abstract:**

Background and Objectives: GLP-1 receptor agonists (GLP-1 RAs) are effective weight-loss therapies, but data on body composition changes in pediatric obesity remain scarce. The primary objective was to evaluate the effects of GLP-1 RAs on body composition in children with obesity. Materials and Methods: We conducted a retrospective study of children with obesity evaluated at the National Institute for Mother and Child Health “Alessandrescu-Rusescu”, Bucharest, Romania, who initiated weekly injectable GLP-1 RA therapy (semaglutide) between January and December 2025. Patients were assessed at baseline and after a median follow-up of 5 months. Eight of ten participants with complete paired data were included in the final analysis; two were excluded because one was a non-responder with weight gain and suspected non-compliance, while one responder could not maintain the standing position for bioimpedance measurement. Bioimpedance analysis and anthropometry were performed at both visits. Paired data were analyzed using Wilcoxon signed-rank tests. Results: Eight children (4 boys, 4 girls; mean age 14.9 ± 1.8 years) completed the study. Significant Body Mass Index (BMI) Z-score improvements were observed (CDC: −0.14, *p* = 0.012; WHO: −0.37, *p* = 0.012), with a median weight reduction of 4.75 kg (*p* = 0.036). While absolute muscle mass showed non-significant change (−1.3 kg, *p* = 0.362), predicted muscle mass percentage increased significantly (+1.9%, *p* = 0.012), suggesting selective fat loss. Fat-free mass percentage increased (+2.0%, *p* = 0.012) with reciprocal fat mass reduction (absolute: −3.85 kg, *p* = 0.017; percentage: −2.0%, *p* = 0.012). Fat-free mass index remained stable (−0.67 kg/m^2^, *p* = 0.161). No serious adverse events occurred. Sensitivity analysis (*n* = 10) confirmed the robustness of the results, with improvements in BMI Z-scores remaining significant. Conclusions: In this preliminary pilot study, GLP-1 RA therapy in children with obesity was associated with significant improvements in BMI Z-scores and favorable shifts in body composition, consistent with selective fat loss and relative preservation of lean mass. These exploratory findings are hypothesis-generating and support the conduct of larger prospective controlled studies with body composition as a primary endpoint.

## 1. Introduction

The incidence and prevalence of obesity are rising among children and young people [1]. This can lead to complications such as hypertension, impaired glucose tolerance, type 2 diabetes mellitus, sleep apnea, hepatic steatosis, and dyslipidemia [2]. Excess adiposity has also been independently associated with impaired muscle strength and reduced functional capacity, further affecting the quality of life of children and adolescents [3]. While lifestyle changes, such as diet and exercise, are essential to combat obesity, they are sometimes not enough. Luckily, new molecules such as GLP-1 receptor agonists (GLP-1 RAs) have been introduced as treatments for obesity, some of which have been cleared for use in children. There is a significant lack of data on the effects of body composition in children. Published studies have focused on weight loss and Body Mass Index (BMI) rather than on muscle mass measured by dual-energy X-ray absorptiometry (DXA) or other precise methods. This research gap is particularly important in pediatric patients, given the importance of muscle development during this period of growth.

GLP-1 RAs mimic the action of the endogenous hormone GLP-1 by enhancing glucose-dependent insulin secretion, by suppressing glucagon release, and by slowing gastric emptying. Also, through hypothalamic pathways, they reduce appetite and promote satiety, the result being a decrease in caloric intake resulting in substantial weight loss. They have a prolonged therapeutic effect compared to the endogenous version because they are resistant to degradation by dipeptidyl peptidase-4 (DPP-4). Tirzepatide extends this pharmacological profile further as a dual agonist, targeting both GLP-1 and GIP receptors. Although they were initially developed to treat type 2 diabetes, GLP-1 RAs have demonstrated potential in aiding weight loss and lowering the risk of cardiovascular diseases [4,5,6]. Three injectable incretin analogs are approved by the FDA for weight management as adjuncts to lifestyle modifications: liraglutide (3.0 mg once daily), semaglutide (up to 7.2 mg once weekly), and tirzepatide (a dual GIP/GLP-1 receptor agonist, up to 15 mg once weekly).

Liraglutide’s effectiveness was evaluated in the SCALE trials, demonstrating an average weight loss of 8.0% versus 2.6% with placebo at 56 weeks [7]. It was also tested in adolescents in the SCALE Teens trial (ages 12–18), achieving an estimated treatment difference in BMI standard deviation score of −0.22 compared to the placebo group [8]. More recently, the SCALE Kids trial (ages 6–12) showed a 5.8% decrease in BMI, whereas the placebo group experienced a 1.6% increase, making liraglutide the first GLP-1 RA studied in children under 12 [9]. Semaglutide (2.4 mg once weekly) showed superior effectiveness in adults within the STEP trial, with an average weight loss of 14.9% at 68 weeks compared with −2.4% placebo [10]. It produced even greater results among adolescents in the STEP Teens trial (ages 12–18), with a mean BMI reduction of 16.1%, compared to 0.6% with placebo [11]. Recently, the FDA approved Semaglutide 7.2 mg/week [12]. The STEP-UP trial demonstrated greater average weight loss with semaglutide 7.2 mg than with 2.4 mg (−18.7% versus −15.6%) or with placebo (−18.7% versus −3.9%) [13]. The 7.2 mg dose has not been evaluated in pediatric populations. Tirzepatide was studied in the SURMOUNT-1 trial, and participants achieved a mean percentage change in weight at week 72 of −15.0% with 5 mg weekly doses, −19.5% with 10 mg doses, and −20.9% with 15 mg doses, compared to −3.1% in the placebo group [14]. In pediatric populations, tirzepatide has only been evaluated in youth-onset type 2 diabetes through the SURPASS-PEDS trial. The results showed, beyond improving HbA1c, a 7.4% and 11.2% BMI reduction in the 5 mg and 10 mg groups, respectively, compared with a 0.4% decrease in the placebo group at 30 weeks [15]. 

GLP-1 RAs promote favorable changes in body composition in adults. DXA analysis from the STEP 1 substudy showed that semaglutide treatment led to notable decreases in total fat mass (−19.3%) and visceral fat (−27.4%), with a smaller drop in lean body mass (−9.7%). Notably, the percentage of lean body mass relative to total body mass rose by 3.0 points, and the lean-to-fat ratio improved considerably [16]. The SURMOUNT-1 DXA substudy demonstrated that tirzepatide leads to even more selective fat loss, with 75% of weight reduction coming from fat mass and 25% from lean mass [17]. Despite decreases in absolute lean mass, the proportion of lean mass relative to total body weight increased with both agents, indicating that metabolically active tissue is preserved during weight loss.

In children, GLP-1 RAs demonstrated efficiency in reducing body weight, BMI, BMI z-score, and waist circumference, but data on actual body composition changes are limited [8,9,11,18]. Most pediatric research depends on indirect measures, such as anthropometric data, rather than direct assessments of fat mass, lean mass, or muscle function, which would require techniques like DXA or bioimpedance analysis. Results from certain studies show that GLP-1 RAs decrease waist circumference, which suggests central fat loss [19,20]. However, the impact on lean mass preservation and muscle function remains unclear in children. This knowledge gap is concerning, as muscle development is crucial during growth. To our knowledge, to date, there is no real-world pediatric study that has systematically evaluated body composition changes with bioelectrical impedance analysis (BIA) during weekly GLP-1 RA treatment.

The primary objective of the current research was to evaluate the effects of GLP-1 RAs on body composition, with a focus on muscle mass, in children with obesity. Second, we evaluated their effect on weight, BMI, and waist and hip circumferences. Additionally, the presence of obesity-related complications, such as insulin resistance, dyslipidemia, altered glycemia or type 2 diabetes, hepatic steatosis, hypertension, and sleep apnea syndrome, was assessed.

Given the limited data available, the present study is a pilot, exploratory investigation intended to generate hypotheses for future larger controlled trials.

## 2. Materials and Methods

### 2.1. Study Design and Participants

We conducted a retrospective longitudinal study of children with obesity who initiated weekly injectable GLP-1 RA therapy between January and December 2025 and who were evaluated in the Pediatric Endocrinology Department at the National Institute for Mother and Child Health “Alessandrescu-Rusescu” in Bucharest, Romania. All parents or legal guardians of children participating in this study signed the informed consent. The research adhered to national regulations and institutional policies, aligning with the Helsinki Declaration, and received approval from the local ethics committee. Participants’ data were introduced into an anonymized database that did not contain personal identifying information.

The inclusion criteria were age between 12 and 18 years at the baseline visit, a diagnosis of obesity (BMI percentile above the 95th), and failure of lifestyle changes, with at least one follow-up visit. Exclusion criteria included genetic or syndromic obesity, secondary causes of obesity, and contraindications to GLP-1 RA treatment. Patients were assessed at baseline (before treatment) and at follow-up, after a median of 5 months.

Ten patients (6 boys and 4 girls, with a median age of 15 years) initiated weekly injectable GLP-1 RA therapy during the study period. Baseline body composition assessment using BIA was successfully completed in all 10 patients. Eight patients with complete paired bioimpedance data at baseline and follow-up were included in the primary analysis of body composition changes. Two patients were excluded from the primary body composition analysis for distinct reasons. The first patient demonstrated weight gain (+3.3 kg) and a substantial reduction in predicted muscle mass percentage (−8.4%) during the observation period. These findings raised concern regarding poor adherence to both pharmacological treatment and lifestyle recommendations. Because the objective of the primary analysis was to evaluate body composition changes during active treatment, this patient was excluded from the paired bioimpedance analysis. Importantly, to evaluate the impact of this exclusion, we performed a sensitivity analysis including all 10 participants, which confirmed persistence of statistically significant improvements in BMI Z-scores. The second patient could not maintain the standing position required for bioimpedance measurement at follow-up despite showing a positive clinical response, with a BMI reduction of −1.6 kg/m^2^. This patient was also included in the sensitivity analyses that did not require follow-up bioimpedance measurements.

### 2.2. Treatment Protocol

Treatment was represented by injectable semaglutide, initiated at 0.25 mg/week. Dose escalation in our clinical practice was guided by therapeutic response: at each monthly visit, the dose was maintained if a reduction in BMI and/or fat mass percentage was observed compared with the previous assessment; otherwise, and provided the dose was well tolerated, the dose was increased to the next step (0.25 → 0.5 → 1.0 → 1.7 → 2.4 mg/week). The median dose at follow-up was 0.5 mg weekly. All patients benefited from lifestyle counseling at each visit as an adjunct to pharmacotherapy, including guidance on a balanced diet with adequate protein intake, a goal of at least 60 min of moderate- to vigorous-intensity physical activity/day, and sleep hygiene appropriate for the pediatric age group.

### 2.3. Clinical and Paraclinical Investigations

At the initial visit, a physical examination was conducted, including anthropometric measurements, assessment of pubertal stage, and blood pressure measurement. Height was measured with a stadiometer with 0.1 cm precision and was expressed as standard deviation (SD) scores using synthetic growth references for the Romanian pediatric population [21]. Weight and body composition were measured simultaneously using the Tanita device (Tanita Corporation, Tokyo, Japan; 0.1 kg precision). BMI was calculated using the formula (body weight [kg]/body height [m]^2^). Percentiles and standard deviations were determined using CDC and WHO growth charts and BMI categories were defined according to CDC growth charts: normal weight (<85th percentile), overweight (85th to <95th percentile), Class 1 obesity (95th percentile to <120% of 95th percentile), Class 2 obesity (120% to <140% of 95th percentile), and Class 3 obesity (≥140% of 95th percentile). Waist circumference was measured at the midpoint between the last rib and the iliac crest, and hip circumference was measured at the maximum circumference over the buttocks. Then, waist-hip and waist-height ratios were determined. The pubertal stage was assessed using Tanner staging.

Before starting GLP-1 RA therapy, baseline laboratory tests were performed to assess metabolic status and screen for complications or contraindications. These investigations included fasting glucose, insulin, and HbA1c to assess glycemic control and insulin resistance, and lipid profile (total cholesterol, LDL-cholesterol, HDL-cholesterol, and triglycerides) to evaluate cardiovascular risk factors. At baseline, additional investigations to assess obesity-related complications were also recommended. Children underwent an abdominal ultrasound to check for hepatic steatosis or possible gallbladder lithiasis. Although polysomnography was recommended for obstructive sleep apnea screening in several patients, this investigation was not performed due to the limited availability of pediatric sleep study facilities.

HOMA-IR was calculated using the formula fasting insulin in μU/mL × fasting glucose in mg/dL divided by 405. A threshold of >2 was set to identify insulin resistance. Additionally, the triglyceride-to-HDL cholesterol ratio (Tg/HDL), a documented marker associated with insulin resistance, atherosclerosis, and increased cardiometabolic risk in adults and children, was also calculated. Since conventional adult cut-offs may underestimate metabolic risk in children, we used lower screening thresholds that have been shown to improve early detection. A Tg/HDL ratio > 1.79 and fasting glucose ≥ 87.5 mg/dL served as indicators for insulin resistance and impaired glucose metabolism, respectively, based on ROC-derived optimal values from a Romanian pediatric obesity cohort [22]. These thresholds help to detect metabolic dysfunctions before serious end-organ damage occurs.

Body composition was assessed by BIA using a TANITA DC-430 body composition analyzer (Tanita Corporation, Tokyo, Japan), both at baseline and at follow-up visits. Measurements were performed according to the manufacturer’s instructions: subjects were measured in the morning after an overnight fast, with an empty bladder, wearing light clothing without shoes or metal accessories. Measurements were performed in a standing position on the device platform [23]. The parameters extracted from the device were predicted muscle mass (PMM), fat-free mass (FFM), fat mass, both in absolute and percent values, total body water, phase angle, and other body composition metrics. FFMI (fat-free mass index) was calculated as FFM (kg)/Height^2^ (m^2^) [24].

At the follow-up visit, the same anthropometric measurements were performed. Body composition was also assessed, and changes in parameters were measured. The percentage change in muscle mass was calculated. Children were assessed for adverse reactions and treatment adherence.

### 2.4. Statistical Analysis

Statistical analysis was conducted using SPSS Version 31.0.1.0 (IBM Corp., Armonk, NY, USA). The primary analysis included eight patients, with paired parameters at baseline and follow-up using the Wilcoxon signed-rank test for paired samples. The reported changes represent the median of within-subject differences (individual patient changes from baseline to follow-up). Age is presented as mean ± standard deviation (SD); all other continuous variables are presented as medians [interquartile range (IQR)]. For categorical variables, data were presented as frequencies and percentages. The sensitivity analysis included all 10 subjects and assessed the effect on BMI Z-score to evaluate the robustness of the results, despite one non-responder and one responder with incomplete bioimpedance data. Effect sizes for Wilcoxon signed-rank tests were calculated as r = |Z|/√n, where Z is the standardized test statistic and *n* is the number of pairs and interpreted as small (r = 0.10–0.29), medium (r = 0.30–0.49), or large (r ≥ 0.50) [25]. To identify predictors of treatment response, Spearman’s rank correlation coefficients were calculated between baseline age and treatment outcomes. The association between fat mass loss and improvement in BMI Z-score was evaluated to identify the primary driver of BMI reduction. A two-sided *p*-value below 0.05 was considered statistically significant for all tests.

### 2.5. Outcomes

The primary outcome was represented by the changes in body composition after GLP-1 RA treatment. Secondary outcomes included the effects on anthropometric measurements (weight, BMI, abdominal circumference, waist circumference, abdominal-to-waist ratio, waist-to-height ratio), screening for obesity-related complications, and assessing safety.

### 2.6. Use of Generative Artificial Intelligence

AI-based tools (Claude Sonnet 4.6—Anthropic, San Francisco, CA, USA, and Grammarly, version 1.2.272.1911; Grammarly Inc., San Francisco, CA, USA) were used to create graphics, for language editing and for formatting of the manuscript. The authors take full responsibility for the accuracy and integrity of all content.

## 3. Results

### 3.1. General Characteristics

**Figure 1:**
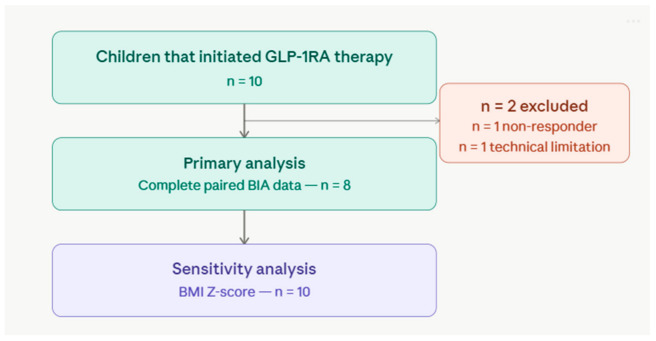
Study Flowchart.

Out of the ten children who initiated GLP-1 RA therapy during the study period, the primary analysis contained eight patients with complete paired data at baseline and follow-up, since two participants were excluded because one demonstrated treatment failure with weight gain and suspected non-compliance, while one responder could not maintain the standing position required for BIA at follow-up. All ten patients were included in a sensitivity analysis regarding BMI Z-score changes to assess the robustness of the findings. The patient flowchart is represented in Figure 1.

The sex distribution was equal (4 boys and 4 girls), with a mean age of 14.9 ± 1.8 years. All patients were in late puberty at baseline, with two patients at Tanner stage 4 and six at Tanner stage 5. Before starting GLP-1 RA treatment, children were screened or referred for evaluation of comorbidities related to obesity. Seven of them had at least one comorbidity. The most frequent comorbidity was insulin resistance, indicated by either a high HOMA-IR index or a high Tg/HDL ratio. Half of the subjects had elevated blood pressure for their age. Other comorbidities detected were dyslipidemia (37.5%), hepatic steatosis (25%), and prediabetes (25%) with HbA1c > 5.6%. Five children had fasting plasma glucose > 85 mg/dL. All initial characteristics are summarized in Table 1.

Individual patient data corresponding to the parameters reported in Table 1, Table 2 and Table 3 are provided in Supplementary Table S1.

### 3.2. Primary Outcome: Body Composition

BIA was performed both at baseline and at follow-up, the median between visits being 5 months. The parameters recorded included fat percentage (FATP), fat mass (FATM), fat-free mass (FFM), total body water (TBW), predicted muscle mass (PMM), body mass index (BMI), water mass (WATERM), water percentage (WATERP), and phase angle, all provided by the Tanita DC430 MA system. Additionally, the following parameters were calculated: percentage muscle mass, percentage fat-free mass, and fat-free mass index (FFMI). The data from the 8 patients with complete paired measurements are detailed in Table 2.

**Figure 2:**
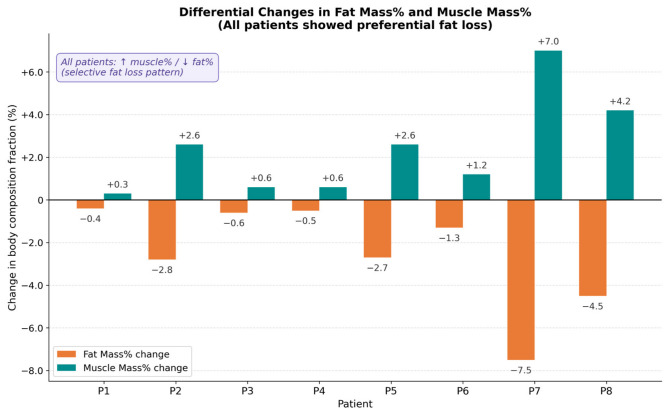
Individual changes in fat mass percentage and predicted muscle mass percentage following GLP-1 RA therapy (*n* = 8). Arrows in the inset annotation indicate the direction of change (↑ = increase, ↓ = decrease).

Although median FFM values were higher at follow-up, the paired within-subject analysis revealed a small non-significant decrease in absolute terms (−1.35 kg, *p* = 0.401), reflecting inter-individual variability. However, FFM increased significantly as a percentage of total body weight (+2.0%, *p* = 0.012). Similarly, predicted muscle mass decreased slightly in absolute terms (−1.3 kg, *p* = 0.362), but increased significantly as a percentage (+1.9%, *p* = 0.012). FFMI remained stable, with a change of −0.67 kg/m^2^ (*p* = 0.161). In contrast, fat mass decreased significantly both in absolute values (−3.85 kg, *p* = 0.017) and as a percentage (−2.0%, *p* = 0.012). Effect sizes for statistically significant parameters were large (r ≥ 0.50). These results indicate targeted fat loss with preservation of lean body mass, as shown by the significant increases in FFM and predicted muscle mass percentages despite minimal changes in their absolute values. This pattern was observed in all eight patients, with fat mass percentage decreasing and predicted muscle mass percentage increasing in every individual case (Figure 2). The phase angle, which is a marker of cellular health and membrane integrity, remained stable throughout treatment (6.1° to 6.2°, *p* = 0.674), suggesting the preservation of cellular function and tissue quality despite significant fat mass reduction. The results regarding BIA are summarized in Table 2.

### 3.3. Anthropometric Changes

Anthropometric outcomes indicated significant efficacy and safety. Weight decreased by a median of 4.75 kg (*p* = 0.036), with corresponding BMI Z-score improvements (CDC BMI Z-score: −0.14, *p* = 0.012; WHO BMI Z-score: −0.37, *p* = 0.012). Importantly, no evidence of impaired linear growth was observed over the follow-up period (+0.3 cm [0.0–1.0], *p* = 0.042). Central adiposity measures showed trends toward improvement, with reductions in waist circumference (−2.5 cm, *p* = 0.127), waist-to-hip ratio (−0.028, *p* = 0.091), and waist-to-height ratio (−0.022, *p* = 0.069), suggesting beneficial effects on body fat distribution, but these decreases did not reach statistical significance. Effect sizes for statistically significant anthropometric parameters were large (r ≥ 0.50). The data on the impact on anthropometric parameters are presented in Table 3.

Beyond statistical measures, 5 out of 8 patients (62.5%) improved their BMI category: one achieved normal weight, three transitioned from Class 1 obesity to overweight, and one improved from Class 3 to Class 2 obesity. Three patients (37.5%) remained in their baseline category despite measurable reductions in BMI.

### 3.4. Correlations of Treatment Response

Age was identified as an important predictor of treatment response (see Table 4). Younger children showed significantly greater improvement in several parameters, including a decrease in BMI Z-score (Spearman’s rho = −0.786, *p* = 0.021), BMI reduction (rho = −0.755, *p* = 0.031), weight loss (rho = −0.738, *p* = 0.037), and fat mass loss (rho = −0.738, *p* = 0.037). Additionally, younger age was associated with a greater increase in predicted muscle mass percentage (rho = 0.643, *p* = 0.086), indicating that younger patients may present better muscle preservation, although this trend did not reach statistical significance.

Furthermore, there was a very strong correlation between fat mass loss and improvement in BMI Z-score WHO (rho = 0.922, *p* = 0.001), indicating that fat reduction was the main factor behind BMI improvements.

### 3.5. Safety

It is important to mention that no severe adverse events were reported during the study. One patient presented with constipation, but this symptom was present before treatment initiation. Another patient reported an increased frequency of intercurrent infections (varicella, hand-foot-and-mouth disease), though a causal relationship with GLP-1 RA therapy could not be established.

### 3.6. Sensitivity Analysis

When we included all 10 patients in the sensitivity analysis, BMI Z-score improvements remained statistically significant (CDC: −0.09, *p* = 0.032; WHO: −0.30, *p* = 0.009), confirming the robustness of the results despite the fact that one child was a non-responder. Effect sizes were large for all parameters in the sensitivity analysis (r ≥ 0.50). Data is presented in Table 5.

## 4. Discussion

There are several key findings from this pilot study regarding the GLP-1 RA therapy in pediatric obesity. First, this therapy led to preferential fat loss rather than uniform weight reduction. While absolute muscle mass had a modest and non-significant decrease, PMM as a percentage of body weight increased significantly. This muscle-sparing effect was also supported by the significant rise in FFM percentage that was mirrored by the decrease in fat mass percentage. Moreover, FFMI showed a minimal and non-significant change, indicating the preservation of lean body mass relative to height. The strong correlation between fat mass loss and BMI Z-score improvement demonstrates that fat reduction, rather than muscle or water loss, primarily led to anthropometric improvements. Second, the extent of BMI Z-score improvement was clinically meaningful. The statistically significant median BMI Z-score reduction indicates a substantial improvement in this pediatric population with severe obesity, in which even modest Z-score decreases are difficult to obtain. Five of eight patients (62%) improved their BMI category, with one reaching a normal weight, indicating real-world clinical benefit beyond statistical significance. Sensitivity analysis, including all 10 patients, confirmed these findings despite one non-responder, highlighting their robustness. Third, the treatment was very well tolerated, with no severe adverse events. This favorable safety profile, together with preserved muscle mass and continued linear growth, supports the use of GLP-1 RA in adolescent populations.

Lean mass reduction during weight loss is a common physiological response to energy deficit, observed across all weight reduction modalities, with the proportion varying according to the degree of caloric restriction, exercise, and type of intervention [26]. Dietary restriction alone is associated with approximately 25% of weight loss originating from fat-free mass, a proportion substantially attenuated when combined with exercise [26,27]. A meta-analysis by Nuijten et al. indicated that pooled lean body mass loss after bariatric surgery was up to −8.13 kg at 12 months postoperatively [28]. In our cohort, during weight loss, FFM and PMM decreased in absolute terms, accounting for approximately 27–30% of total weight reduction. These proportions are consistent with the range of lean mass losses reported in adult DXA studies during GLP-1 RA therapy: approximately 40% with semaglutide (STEP-1) and approximately 25% with tirzepatide (SURMOUNT-1) [10,17]. Although the parameters assessed by BIA and DXA are different, the similar proportional losses indicate that BIA might be useful for clinical tracking during pediatric GLP-1 RA treatment. Similar to the results from the STEP 1 DXA substudy, where lean body mass proportion increased by 3% despite overall decreases, we noticed a 2% rise in FFM and a 1.9% increase in PMM relative to body weight, even with non-significant declines in absolute values. This pattern, where weight loss results primarily from fat reduction while maintaining FFM and muscle tissue proportions, indicates positive body composition changes and appears consistent in pediatric and adult populations.

Body composition has not been analyzed in large pediatric GLP-1 RA trials. A retrospective study by Apperley et al. examined body composition among other parameters during GLP-1 RA therapy in 24 adolescents with severe obesity treated with liraglutide for three months [29]. Results indicated a reduction of 4 kg in absolute fat mass and a 2.09% decrease in body fat percentage, consistent with our findings. Remarkably, our research focused on once-weekly agents, which may lead to improved adherence compared with daily liraglutide, especially in children, where compliance is influenced by the treatment burden. Additionally, Apperley et al. observed a nonsignificant increase in absolute FFM, but did not assess lean mass as a percentage of body weight or report the fat-free mass index. Our study extends these observations by suggesting that, when expressed relative to total body weight, both PMM percentage and FFM percentage increase significantly, with absolute fat-free mass remaining stable. This is particularly relevant in pediatric care.

Our findings show similar efficiency to the STEP TEENS trial. Although STEP TEENS reported a 16.1% BMI decrease over 68 weeks [11], our cohort experienced a 4.87% reduction over only 5.4 months (~0.9% per month). This suggests comparable treatment response rates in real-world settings.

The difference in treatment response related to age is a significant clinical finding with important implications in therapeutic choices. Younger children showed notably better outcomes than older adolescents, with more important effects across all obesity-related parameters. This trend was also observed in a post hoc analysis of the STEP TEENS trial, which found that younger (12–15 years old) versus older (15–18 years old) adolescents achieved greater BMI improvements [30]. This age trend may reflect several factors: (1) earlier intervention before more resistant metabolic issues develop; (2) possibly higher GLP-1 receptor sensitivity in younger individuals; (3) age-related differences in metabolic rate and energy expenditure independent of pubertal stage, given that all patients in this cohort were in late puberty; or (4) behavioral factors such as adherence to treatment and diet. The observed tendency for improved muscle preservation in the younger group further indicates the benefits of early intervention.

These findings have several practical implications for clinical management. The pattern of preferential fat loss with relative preservation of PMM percentage is encouraging. However, it is important to promote adequate protein intake and resistance exercises as supportive measures alongside pharmacotherapy to prevent muscle loss during weight loss [26,27]. The strong negative correlation between age and treatment response indicates that starting GLP-1 RA therapy early may lead to better outcomes, aligning with post hoc analyses of the STEP TEENS trial [30]. Furthermore, because GLP-1 RA therapy significantly suppresses appetite, clinicians should actively monitor for excessive dietary restriction and disordered eating behaviors throughout treatment, including symptoms of avoidant/restrictive food intake disorder (ARFID), particularly in adolescents undergoing rapid weight loss [31,32].

As strengths of the current study, we would like to highlight the longitudinal design and the use of real-world data. Another plus of this study is the assessment of body composition changes using bioimpedance analysis. While most large pediatric GLP-1 RA trials have focused on anthropometric outcomes (weight, BMI, waist circumference), detailed body composition data, including fat mass, muscle mass, and fat-free mass, have been limited in the pediatric literature. To our knowledge, this is the first study to systematically assess changes in body composition using bioimpedance in children treated with weekly injectable GLP-1 RAs. While DXA remains the gold standard for body composition assessment, BIA offers several practical advantages in clinical settings: it is fast, non-invasive, radiation-free, and provides immediate results, making it particularly suitable for serial monitoring in pediatric populations. Another strength of the study is that we performed a sensitivity analysis including all enrolled patients (*n* = 10), regardless of treatment response or completeness of bioimpedance data, to assess BMI Z-score changes. This confirmed the robustness of our anthropometric findings despite one non-responder/non-compliant with weight gain. Also, we would like to emphasize the study’s real-world clinical setting as a strength, reflecting achievable outcomes outside controlled trial conditions.

One of the limitations of this study was its retrospective design and single-center setting with a limited number of participants. The observational, non-randomized design introduces potential confounders, including variability in adherence to lifestyle interventions, dietary habits, and physical activity. Prospective standardization of these co-interventions will be essential in future studies. The pilot nature of the study precludes definitive conclusions and limits statistical power. The sample size does not allow subgroup analyses or multivariable modeling. These data are intended to inform the design and power calculation of a future adequately powered prospective trial. The results warrant a larger prospective study with body composition as the primary outcome, but the number of patients is limited by the medication’s cost, which is not reimbursed. The TANITA DC-430 device used in the present study is part of a series that has been shown to offer the most accurate body composition estimates compared with DXA among BIA methods in children and adolescents with obesity [33]. However, BIA has inherent limitations, including assumptions about hydration status and tissue conductivity, which may affect accuracy in severely obese individuals. Furthermore, the muscle tissue is not measured directly, but results from a prediction, so BIA-derived muscle mass should be interpreted carefully, as changes may reflect shifts in hydration and body composition and not true alterations in skeletal muscle mass. One patient was unable to complete the follow-up BIA evaluation due to difficulty in maintaining the standing position required by the device. This highlights a practical limitation of standing bioimpedance platforms in extremely obese pediatric patients and suggests the need for alternative assessment methods for patients unable to use standard equipment. Future studies should incorporate DXA as a co-primary or confirmatory measurement to validate BIA-derived body composition estimates. One patient with suspected non-compliance was excluded, highlighting the importance of adherence in pediatric populations. Another limitation of the study was the lack of a control group, which hampers the ability to determine causality for the observed changes. Without a concurrent control group, it is not possible to definitively attribute body composition changes to GLP-1RA therapy rather than to lifestyle intervention alone or natural growth trajectories. Further studies may consider controlled real-world comparisons, such as matched lifestyle-only cohorts. Another limitation is the short follow-up period, which is insufficient to assess long-term body composition trajectories, the durability of treatment effects, or potential rebound effects. A longer follow-up is a priority for future studies.

Future directions include extending the follow-up period and enrolling a larger cohort of patients to better characterize the long-term evolution of metabolic and body composition parameters. We also aim to evaluate whether the observed treatment effects are sustained after discontinuation of GLP-1 RA therapy. Additionally, we plan to compare outcomes in this cohort with those of eligible patients who did not wish to pursue pharmacological treatment or could not afford it and instead pursued lifestyle intervention alone, thereby providing a valuable real-world comparison group.

## 5. Conclusions

In this pilot study, GLP-1 RA therapy in pediatric obesity was associated with significant improvements in BMI Z-scores and favorable shifts in body composition, consistent with selective fat loss and relative lean mass preservation, even prior to reaching maximum approved doses. These preliminary findings are hypothesis-generating and support the conduct of larger prospective controlled trials with body composition as a primary endpoint.

## Figures and Tables

**Table 1:** Baseline characteristics of participants.

Characteristic	Value (*n* = 8)
Age (years), mean ± SD	14.9 ± 1.8
Sex, *n* (%)	
Male	4 (50%)
Female	4 (50%)
Tanner stage, *n* (%)	
Stage 4, *n* (%)	2 (25%)
Stage 5, *n* (%)	6 (75%)
Height (cm), median [IQR]	162.8 [154–177.7]
Weight (kg), median [IQR]	79.3 [74.2–130.2]
BMI (kg/m^2^), median [IQR]	31.2 [28.8–41.2]
BMI Z-score CDC, median [IQR]	2 [1.7–2.7]
BMI Z-score WHO, median [IQR]	2.6 [2.1–4.1]
Obesity-related comorbidities, *n* (%)	7 (87.5%)
Insulin resistance	7 (87.5%)
Dyslipidemia	3 (37.5%)
Hypertension/high blood pressure	4 (50%)
Hepatic steatosis	2 (25%)
Prediabetes	2 (25%)
HbA1c (%), median [IQR]	5.4 [5.3–5.6]

**Table 2:** The impact on body composition parameters.

Parameter	Baseline	Follow-Up	Change	*p*	r
FFM (kg)	52.60 [44.50–80.63]	54.60 [43.00–77.65]	−1.35 [−3.38–0.88]	0.401	0.297
FFM (%)	63.9 [59.4–66.8]	64.6 [60.9–72.9]	+2.0 [0.5–4.0]	0.012 *	0.891
FFMI (kg/m^2^)	19.85 [18.38–25.40]	20.20 [17.72–24.24]	−0.67 [−1.44–0.35]	0.161	0.495
PMM (kg)	49.95 [42.27–76.70]	51.85 [40.80–73.80]	−1.30 [−3.27–0.88]	0.362	0.322
PMM (%)	60.7 [56.4–63.5]	61.3 [57.9–69.2]	+1.9 [0.6–3.8]	0.012 *	0.891
Fat mass (kg)	31.30 [23.05–49.32]	28.45 [18.82–45.97]	−3.85 [−5.23 to −1.45]	0.017 *	0.841
Fat mass (%)	36.1 [33.2–40.6]	35.4 [27.0–39.0]	−2.0 [−4.1 to −0.5]	0.012 *	0.891
Phase angle (°)	6.1 [5.5–7.8]	6.2 [5.9–7.0]	−0.2 [−0.6–0.4]	0.674	0.148

Data presented as medians [IQR]. Change represents the median of paired individual differences. * *p* < 0.05 (Wilcoxon signed-rank test).

**Table 3:** Anthropometric changes during the study period.

Parameter	Baseline	Follow-Up	Change	*p*	r
Weight (kg)	79.30 [74.25–130.20]	78.70 [69.23–121.95]	−4.75 [−10.13 to −0.08]	0.036 *	0.742
Height (cm)	162.8 [154.0–177.8]	164.3 [154.3–178.4]	+0.3 [0.0–1.0]	0.042 *	0.718
BMI (kg/m^2^)	31.20 [28.80–41.28]	30.20 [26.78–38.98]	−1.95 [−3.15 to −0.80]	0.017 *	0.842
BMI % change	-	-	−4.87% [−9.10 to −2.62]	0.017 *	
BMI Z-score (CDC)	2.07 [1.72–2.74]	1.93 [1.43–2.66]	−0.14 [−0.34 to −0.07]	0.012 *	0.891
BMI Z-score (WHO)	2.70 [2.12–4.10]	2.45 [1.69–3.74]	−0.37 [−0.54 to −0.21]	0.012 *	0.892
Waist circumference (cm)	88.0 [82.5–117.3]	86.5 [80.5–113.5]	−2.5 [−6.8 to −0.5]	0.127	0.54
Hip circumference (cm)	106.0 [100.3–127.0]	109.0 [101.3–126.6]	0.0 [−2.0 to 1.0]	0.752	0.112
Waist-to-hip ratio	0.844 [0.790–0.884]	0.805 [0.766–0.901]	−0.028 [−0.055 to −0.002]	0.091	0.598
Waist-to-height ratio	0.564 [0.508–0.684]	0.536 [0.504–0.645]	−0.022 [−0.050 to −0.004]	0.069	0.643

Data presented as medians [IQR]; * *p* < 0.05 (Wilcoxon signed-rank test).

**Table 4:** Age as Predictor of Treatment Response.

Outcome Parameter	Spearman Rho	*p*-Value
BMI Z-score (CDC) reduction	−0.786	0.021 *
BMI reduction	−0.755	0.031 *
Weight loss	−0.738	0.037 *
Fat mass loss	−0.738	0.037 *
PMM % increase	0.643	0.086

* *p* < 0.05 (statistically significant).

**Table 5:** Sensitivity analysis on BMI, including all 10 subjects.

Parameter	Baseline	Follow-Up	Change	*p*	r
BMI (kg/m^2^)	33.7 [29.4–43.95]	33.5 [26.9–40.8]	−1.30 [−3 to −0.8]	0.028 *	0.693
BMI Z-score (CDC)	2.43 [1.75–2.84]	2.39 [1.5–2.73]	−0.09 [−0.2 to −0.01]	0.032 *	0.677
BMI Z-score (WHO)	3.23 [2.16–4.3]	3.21 [1.8–3.91]	−0.30 [−0.49 to −0.16]	0.009 *	0.823

Data presented as medians [IQR]; * *p* < 0.05 (Wilcoxon signed-rank test).

## Data Availability

The raw data can be obtained on request from the corresponding author.

## Supplementary Materials

The following supporting information can be downloaded at https://www.mdpi.com/article/10.3390/metabo16070460/s1, Table S1: Individual patient data.
