# Supplementary material for: Body Composition Changes During GLP-1 Receptor Agonist Therapy in Pediatric Obesity: A Pilot Study
Source: Metabolites. 2026 Jul 1;16(7):460. doi: 10.3390/metabo16070460 (PMC13414465; doi:10.3390/metabo16070460)
Supplement: Supplementary file 1 [file metabolites-16-00460-s001.zip › metabolites-4346147-supplementary.pdf]

Supplementary Table S1. Individual Patient Data (n = 8)

Parameter	P1	P2	P3	P4	P5	P6	P7	P8
**Demographics**								
**Sex**	M	F	M	F	F	M	F	M
**Age, baseline (years)**	15.1	15.8	12.7	11.8	16.5	15.4	17.0	14.7
**Tanner stage**	5	5	4	5	5	5	5	4
**HbA1c (%)**	5.8	5.4	5.9	5.2	5.3	5.5	5.4	5.2
**Anthropometrics (baseline → follow-up)**								
**Height (cm)**	180.8→180.8	158.0→158.0	171.0→172.1	152.6→152.6	161.4→161.7	180.0→180.5	152.7→153.0	164.2→167.0
**Weight (kg)**	141.9→131.3	74.7→67.5	95.1→93.9	81.2→82.3	77.4→75.1	147.6→138.9	66.4→55.1	74.1→74.4
**BMI (kg/m²)**	43.4→40.2	29.9→27.0	32.5→31.7	34.9→35.3	29.7→28.7	45.6→42.6	28.5→23.5	27.5→26.7
**BMI Z-score (CDC)**	2.82→2.71	1.77→1.39	2.36→2.29	2.50→2.49	1.70→1.54	2.90→2.82	1.52→0.64	1.77→1.57
**BMI Z-score (WHO)**	4.30→3.83	2.19→1.63	3.20→2.99	3.50→3.47	2.11→1.90	4.59→4.15	1.86→0.70	2.16→1.86
**Waist circumference (cm)**	130.0→120.0	91.0→83.0	97.0→94.0	85.0→90.0	84.0→82.0	124.0→121.0	77.0→77.0	82.0→80.0
**Hip circumference (cm)**	131.0→129.0	105.0→105.0	115.0→113.0	107.0→119.5	99.0→100.0	140.0→131.0	99.0→99.0	104.0→105.0
**Waist-to-hip ratio**	0.990→0.930	0.867→0.790	0.840→0.832	0.794→0.753	0.848→0.820	0.890→0.924	0.778→0.778	0.788→0.762
**Waist-to-height ratio**	0.720→0.664	0.576→0.525	0.570→0.546	0.557→0.590	0.520→0.507	0.720→0.670	0.504→0.503	0.499→0.479
**Body Composition (baseline → follow-up)**								
**Fat mass (kg)**	52.8→48.3	29.3→24.6	33.3→32.3	38.9→39.0	25.6→22.8	60.7→55.3	22.2→14.3	20.7→17.5
**Fat mass (%)**	37.2→36.8	39.2→36.4	35.0→34.4	47.9→47.4	33.1→30.4	41.1→39.8	33.4→25.9	28.0→23.5
**PMM (kg)**	84.8→78.9	43.1→40.7	58.7→58.5	40.1→41.1	49.2→49.7	82.7→79.5	42.0→38.7	50.7→54.0
**PMM (%)**	59.8→60.1	57.7→60.3	61.7→62.3	49.4→49.9	63.6→66.2	56.0→57.2	63.2→70.2	68.4→72.6
**FFM (kg)**	89.1→83.0	45.4→42.9	61.8→61.6	42.3→43.3	51.8→52.3	86.9→83.6	44.2→40.8	53.4→56.9
**FFM (%)**	62.8→63.2	60.8→63.6	65.0→65.6	52.1→52.6	66.9→69.6	58.9→60.2	66.6→74.0	72.1→76.5
**FFMI (kg/m²)**	27.26→25.39	18.19→17.18	21.13→20.80	18.16→18.59	19.88→20.00	26.82→25.66	18.96→17.43	19.81→20.40
**Phase angle (°)**	5.47→5.92	5.35→5.27	5.70→6.14	6.26→5.90	8.18→7.49	8.26→7.19	6.50→6.22	5.96→6.36

PMM = Predicted Muscle Mass; **FFM** = Fat-Free Mass; **FFMI** = Fat-Free Mass Index; **WHO** = World Health Organization; **CDC** = Centers for Disease Control and Prevention.
